# Manganese Phthalocyanine-Based Magnetic Core–Shell Composites with Peroxidase Mimetic Activity for Colorimetric Detection of Ascorbic Acid and Glutathione

**DOI:** 10.3390/molecules30071484

**Published:** 2025-03-27

**Authors:** Junchao Qi, Long Tian, Yudong Pang, Fengshou Wu

**Affiliations:** Hubei Key Laboratory of Novel Reactor and Green Chemical Technology, Key Laboratory of Novel Biomass-Based Environmental and Energy Materials in Petroleum and Chemical Industry, School of Chemical Engineering and Pharmacy, Wuhan Institute of Technology, Wuhan 430072, China

**Keywords:** phthalocyanine, nanoenzymes, peroxidase, magnetic, colorimetric

## Abstract

Ascorbic acid (AA) and glutathione (GSH) play a pivotal role in health assessment, drug development, and quality control of nutritional supplements. The development of a new and efficient method for their detection is highly desired. In this work, we fabricated magnetic core–shell nanocomposites (Fe_3_O_4_@MnPc-NDs) by a one-pot hydrothermal method with citric acid and manganese tetraamino phthalocyanine (MnTAPc) as precursors. Fe_3_O_4_@MnPc-NDs exhibited enhanced peroxidase activity compared to bare Fe_3_O_4_ nanoparticles, enabling catalytic oxidation of colorless 3,3′,5,5′-tetramethylbenzidine (TMB) to blue ox-TMB in the presence of H_2_O_2_. Leveraging the antioxidant properties of AA/GSH to reduce ox-TMB, a colorimetric assay achieved a low detection limit of 0.161 μM for AA and 0.188 μM for GSH with broad linear ranges. Moreover, this method displayed high specificity against 12 interfering substances and excellent recyclability (>90% activity after five cycles). Finally, the Fe_3_O_4_@MnPc-NDs could act as an efficient colorimetric sensor for accurately detecting AA in genuine VC tablets and GSH in whitening serums with high accuracy. Therefore, Fe_3_O_4_@MnPc-NDs exhibited great potential in bioassay applications, benefiting from their outstanding sensitivity and high recycling rate.

## 1. Introduction

Ascorbic acid, commonly known as vitamin C (AA), holds a pivotal position as a vital vitamin and potent antioxidant in human nutrition, essential for disease prevention and enhancement of immune defenses [1,2,3]. Similar to AA, glutathione (GSH) plays an important role in providing immunity, enhancing antioxidant function, removing free radicals to delay aging [4,5,6,7], and reducing stress and a broad range of ailments, including Parkinson’s disease, Alzheimer’s disease, HIV, diabetes, and cancer [8,9,10,11,12]. However, it is commonly acknowledged that the inherent concentration of AA/GSH in the human body often falls short of fulfilling the requirements for optimal physiological functions, thereby posing a heightened risk of developing various diseases when their levels are depleted. Humans need to consume AA/GSH from their daily diet or functional foods to maintain health. Therefore, to provide guidance for selecting foods, it is of great significance to develop effective and quantitative methods for the determination of AA and GSH [13,14,15,16].

Based on their antioxidant effects, it is feasible to realize the detection of ascorbic acid and glutathione through specific oxidative reactions [17,18]. Among the diverse measurement techniques, colorimetric approaches harnessing peroxidase-mimicking activity have garnered significant attention due to their swiftness, cost-efficiency, and user-friendliness [19,20,21,22]. Horseradish peroxidase [23] is a natural peroxidase that has some inherent disadvantages, such as being time-consuming to extract, easy to inactivate, and difficult to preserve, thus leading to limited practical applications [24]. Therefore, it was imminent to develop potential peroxidase-mimicking enzymes to realize efficient detection. Fe_3_O_4_ exhibited catalytic activity that was analogous to that of horseradish peroxidase, demonstrating its potential as a peroxidase mimic for various applications [25,26,27]. Its preparation into nanoparticles endowed them with peroxidase properties as well as unique properties such as magnetism, low toxicity, and high biocompatibility, which were highly promising for applications [28,29]. Due to the distinctive macrocyclic conjugated aromatic structure, porphyrins have garnered extensive attention in numerous fields, including biomimetics, pharmaceutical science, medicinal chemistry, catalysis, materials chemistry, and coordination chemistry [30,31]. Metal porphyrin compounds were widely present in living organisms in nature and played an important role in life activities, among which horseradish peroxidase was composed of iron porphyrin. The structure of phthalocyanine was similar to porphyrin and belonged to porphyrin derivatives with better catalytic activity and stability [32,33]. Therefore, the combination of metal phthalocyanine with Fe_3_O_4_ might provide exceptional applications in the domains of artificial enzyme mimicry, biosensing platforms, and electrochemical catalysis.

The aim of this study is to develop a reusable magnetic core–shell nanocomposite (Fe_3_O_4_@MnPc-NDs) with enhanced peroxidase-like activity for the dual detection of AA and GSH in complex matrices. By integrating manganese phthalocyanine MnPc with Fe_3_O_4_ nanoparticles through a one-pot hydrothermal synthesis, we address the limitations of natural enzymes (e.g., instability and high cost) and existing nanozymes (e.g., low catalytic efficiency and poor specificity). Through systematic characterization of structural properties and kinetic analysis, this work elucidates the synergistic mechanism between MnPc and Fe_3_O_4_, which significantly improves the catalytic oxidation of TMB in the presence of H_2_O_2_. Furthermore, we establish a colorimetric platform leveraging the antioxidant properties of AA/GSH to reduce ox-TMB, achieving ultra-low detection limits of 0.161 μM for AA and 0.188 μM for GSH, respectively. Finally, the colorimetric method based on Fe_3_O_4_@MnPc-NDs was used to determine the AA content in vitamin C tablets and the GSH content in whitening serum with high precision and reliability.

## 2. Experimental Sections

### 2.1. Synthesis of Manganese Tetranitro Phthalocyanine

**Figure 1:**
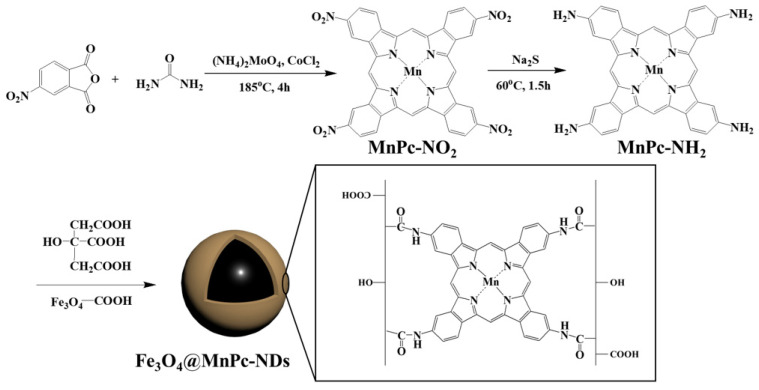
Schematic diagram of the formation of Fe_3_O_4_@MnPc-NDs with core–shell structure.

As shown in Figure 1, a 6.0 g amount (100 mmol) of urea, 4.0 g (20 mmol) of 4-nitrophthalic anhydride, and 656 mg (5.2 mmol) of manganese chloride were dissolved in 30 mL of nitrobenzene solution. Afterward, 26 mg (0.02 mmol) of ammonium molybdate was added to the reaction mixture, which was then heated at 185 °C for 4 h under a nitrogen atmosphere. The reaction mixture was then cooled to room temperature and diluted with toluene. The suspension was centrifuged to collect the formed sediment, which was washed thoroughly with toluene, water, methanol/ether (1:9), and ethyl acetate/hexane (2:1) successively. Finally, the solid was dried under vacuum to obtain manganese tetranitro phthalocyanine (MnPc-NO_2_) in 80% yield.

### 2.2. Synthesis of Manganese Tetraamino Phthalocyanine

A 7.4 g amount (30.9 mmol) of sodium sulfide nonahydrate and 1.92 g (2.5 mmol) of manganese tetranitro phthalocyanine were dissolved in 50 mL of DMF. Under nitrogen atmosphere, the reaction mixture was heated to 60 °C and agitated for a period of 1.5 h. After cooling to ambient temperature, the reaction solution was diluted with 150 mL of water. The formed precipitate was then separated by centrifugation, followed by washing with methanol/ether mixture (1:9) and ethyl acetate successively. Finally, the solid was dried under vacuum to obtain manganese tetranitro phthalocyanine (MnPc-NH_2_) in 75% yield.

### 2.3. Synthesis of Magnetic Fe_3_O_4_ Nanoparticles

Magnetic Fe_3_O_4_ nanoparticles were fabricated using the solvothermal approach. Firstly, 0.75 g of PSSMA was dissolved in 20 mL of ethylene glycol and stirred for 15 min. Then 0.8 g of FeCl_3_·6H_2_O and 2.25 g of magnesium acetate were added sequentially and reacted for another 15 min. The reaction mixture was then carefully transferred to a hydrothermal reactor and heated at 200 °C for a duration of 10 h. Upon completion of the reaction, the resultant material was thoroughly rinsed three times using deionized water and anhydrous ethanol, to ensure the removal of impurities. Finally, the material was dried under vacuum for 12 h to yield the desired powder.

### 2.4. Synthesis of Fe_3_O_4_@MnPc-NDs

Firstly, 50 mg of Fe_3_O_4_ nanoparticles were dispersed by sonication in 10 mL of ultrapure water for 10 min. Then 15 mg of manganese tetraamino phthalocyanine and 150 mg of citric acid were sequentially added. After sonication for 10 min, the reaction solution was transferred to a hydrothermal reactor. After heated at 200 °C for 4 h, the resulting solid was separated and washed three times with deionized water and anhydrous ethanol, respectively. Finally, the desired composites were dried under vacuum to obtain the magnetic Fe_3_O_4_@MnPc-NDs with core–shell structure.

### 2.5. Peroxidase Like Catalytic Activity of Fe_3_O_4_@MnPc-NDs

To assess the peroxidase-like properties of Fe_3_O_4_@MnPc-NDs, the catalyzed oxidation of TMB by H_2_O_2_ was determined. In brief, TMB (5 mM, 50 μL) and Fe_3_O_4_@MnPc-NDs (1 mg/mL, 20 μL) were added into HAc-NaAc buffer (pH = 4.0, 2.8 μL). The combined solution was maintained at 25 °C for 7 min. Fe_3_O_4_@MnPc-NDs were then separated from the solution using an external magnet, followed by the measurement of the maximum absorbance at 652 nm.

### 2.6. Kinetic Measurements

The kinetic analysis of Fe_3_O_4_@MnPc-NDs was evaluated by Michaelis–Menten model [18]. In brief, different concentrations of H_2_O_2_ or TMB were added into a HAc-NaAc buffer solution (pH 3.8) containing Fe_3_O_4_@MnPc-NDs (1 µg/mL, 20 μL). The intensity of absorption at 652 nm was monitored at room temperature over time. $k_{m}$ is the Michaelis constant, $v_{0}$ and $v_{m}$ are the initial and maximum reaction velocities of the reaction, respectively, and $\left[s\right]$ is the concentration of the substrate. The computation of this value is carried out using the Michaelis–Menten equation (Equation (1)).

(1) $$\frac{1}{v_{0}}=\frac{k_{m}}{v_{m}\left[s\right]}+\frac{1}{v_{m}}$$

where v_0_ and v_m_ represent the initial rate and maximum rate, respectively. k_m_ stands for the Michaelis–Menten constant and [s] stands for substrate (H_2_O_2_ or TMB) concentration.

### 2.7. Detection of H_2_O_2_ by the Colorimetric Method

A colorimetric detection method for H_2_O_2_ was established based on the relationship between the concentration of H_2_O_2_ and the absorbance of system at 652 nm. Specifically, 50 μL of different concentrations of H_2_O_2_, 20 μL of Fe_3_O_4_@MnPc-NDs suspension (1.0 mg/mL), and 50 μL of TMB solution (5 mM) were added to 2800 μL of HAc-NaAc buffer solution (pH 3.8), which was stirring at 25 °C for 7 min. Fe_3_O_4_@MnPc-NDs were then isolated from the solution by magnet, followed by the measurement of the absorbance at 652 nm. To further investigate the specificity of this method, interfering substances such as ascorbic acid (AA), citric acid (CA), dopamine (DA), Na^+^, K^+^, Ca^2+^, and glucose were selected as control samples.

### 2.8. Determination of AA and GSH by the Colorimetric Method

A colorimetric detection platform was devised based on the antioxidant activity of AA/GSH. Specifically, 20 μL of suspension containing Fe_3_O_4_@MnPc-NDs (1.0 mg/mL), 50 μL of H_2_O_2_ (0.1 M), 50 μL of TMB solution (5 mM), and 50 μL of AA (or GSH) of different concentrations were sequentially added to 2830 μL of HAc-NaAc buffer solution (pH 3.8). After stirring at 25 °C for 7 min, the absorbance at 652 nm was recorded by UV–vis absorption spectrum. The AA or GSH content was measured by observing the variation in absorbance at 652 nm. The selectivity of AA/GSH was determined by comparing the variations in absorbance at 652 nm between AA/GSH and twelve distinct interfering substances (Arg (arginine), His (histidine), Lys (lysine), Gly (glycine), Ala (alanine), Phe (phenylalanine), Val (valine), Mg^2+^, Zn^2+^, Na^+^, K^+^, and Ca^2+^). The concentration of AA/GSH was 60 μM, while the concentrations of the interfering substances were set at a level tenfold higher than that of AA/GSH.

### 2.9. Identification of AA and GSH Concentrations in Real Samples

To explore the practicality of the colorimetric method, a certain weight of vitamin C tablets was dissolved in water and further adjusted to the required volume to determine the amount of AA in vitamin C tablets. For accuracy verification, a standard AA solution was spiked into the diluted vitamin C tablet sample, and the absorbance variation at 652 nm upon vitamin C tablet addition was documented, respectively. Meanwhile, whitening serum was obtained from a nearby beauty shop and the spiked recovery method was employed to quantify the glutathione level in the sample.

## 3. Results and Discussion

### 3.1. Characterization of Fe_3_O_4_@MnPc-NDs

**Figure 2:**
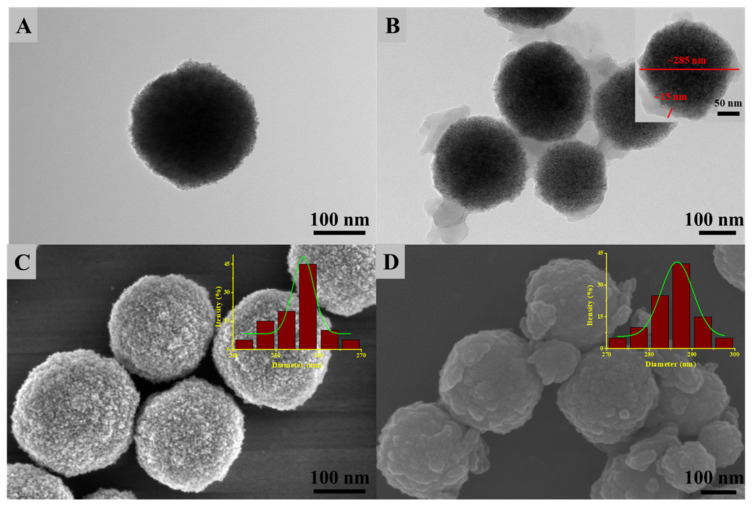
(**A**) TEM images showcasing the morphology of Fe_3_O_4_ nanospheres (**A**), TEM of Fe_3_O_4_@MnPc-NDs (**B**); FESEM visuals revealing the surface details of Fe_3_O_4_-COOH nanospheres (**C**), TEM of Fe_3_O_4_@MnPc-NDs (**D**).

The size and morphology of Fe_3_O_4_ nanospheres and Fe_3_O_4_@MnPc-NDs were characterized by scanning electron microscopy (SEM) and transmission electron microscopy (TEM). As indicated in Figure 2A, the size of Fe_3_O_4_ nanoparticles was about 257 nm. After functionalization with manganese phthalocyanine, the composites with core–shell structures were formed along with the increase in particle size to 285 nm (Figure 2B). The thickness of the shell layer in Fe_3_O_4_@MnPc-NDs was approximated to be 15 nm. FESEM (Figure 2C,D) demonstrated that the surface of Fe_3_O_4_@MnPc-NDs was rougher than that of bare Fe_3_O_4_ nanoparticles, further indicating the successful modification of phthalocyanine to the Fe_3_O_4_ surface.

**Figure 3:**
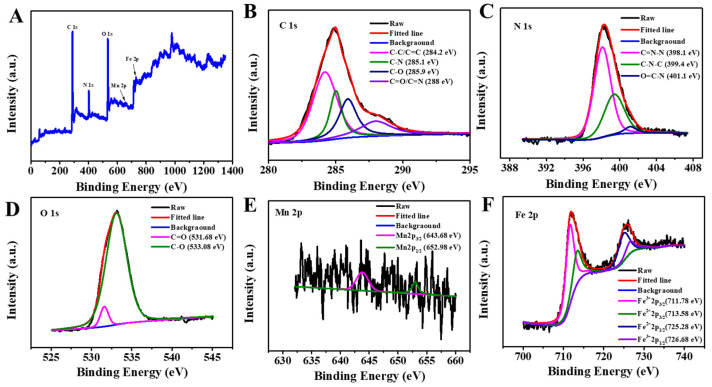
(**A**) XPS spectrum of Fe_3_O_4_@MnPc-NDs; (**B**) C 1s spectrum of Fe_3_O_4_@MnPc-NDs; (**C**) N 1s spectrum of Fe_3_O_4_@MnPc-NDs; (**D**) O 1s spectrum of Fe_3_O_4_@MnPc-NDs; (**E**) Mn 2p spectrum of Fe_3_O_4_@MnPc-NDs; (**F**) Fe 2p spectrum of Fe_3_O_4_@MnPc-NDs.

The elemental composition of Fe_3_O_4_@MnPc-NDs was confirmed by XPS analysis. As indicated in Figure 3A, the distinct peaks at 285, 400, 531, 643, and 726 eV were observed, attributable to C 1s, N 1s, O 1s, Mn 2p, and Fe 2p, respectively. The XPS analysis at high resolution for C 1s orbital (Figure 3B) unveiled four discrete peaks positioned at 284.2, 285.1, 285.9, and 288.0 eV, each corresponding to C-C/C=C, C-N, C-O, and C=O/C=N bonds, respectively. The high-resolution XPS spectrum of N 1s (Figure 3C) showed C=N (398.1 eV) attributed to nitrogen of phthalocyanine ring, C-N (399.4 eV) attributed to the coordination of manganese and nitrogen, and O=C-N (401.1 eV) attributed to amide on the of nitrogen. The XPS analysis at high resolution for O 1s (Figure 3D) showed two peaks at 533.69 and 533.08 eV, attributed to C=O and C-O, respectively. The XPS spectra of Mn 2p could be fitted into two peaks at 652.98 and 643.68 eV, attributable to Mn 2p_1/2_ and Mn 2p_3/2_, respectively, indicating the presence of trivalent manganese ions in Fe_3_O_4_@MnPc-NDs (Figure 3E). Four peaks were observed in the high-resolution XPS spectra of Fe 2p (Figure 3F), matching Fe^2+^ 2p_3/2_ (713.58 eV), Fe^2+^ 2p_1/2_ (725.28 eV), Fe^3+^ 2p_3/2_ (711.78 eV), and Fe^3+^ 2p_1/2_ (726.68 eV) of Fe_3_O_4_ nanoparticles, respectively. Thus, the XPS spectrum clearly confirmed the presence of Fe and Mn elements in Fe_3_O_4_@MnPc-NDs in addition to C, N, and O.

**Figure 4:**
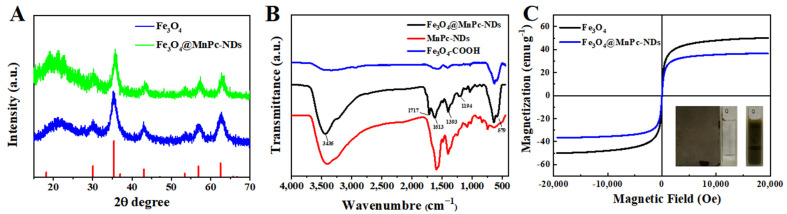
(**A**) XRD patterns of Fe_3_O_4_ and Fe_3_O_4_@MnPc-NDs; (**B**) FT-IR spectra of Fe_3_O_4_, MnPc-NDs, and Fe_3_O_4_@MnPc-NDs; and (**C**) magnetic hysteresis curve of Fe_3_O_4_ and Fe_3_O_4_@MnPc-NDs.

The crystal structures of Fe_3_O_4_ nanoparticles and Fe_3_O_4_@MnPc-NDs were characterized through an X-ray diffraction (XRD) curve. As shown in Figure 4A, the XRD spectrum of Fe_3_O_4_ nanoparticles exhibited distinct diffraction peaks positioned at angles of 30.1°, 35.5°, 43.2°, 53.5°, 57.3°, and 62.9°, attributed to the characteristic lattice planes of (220), (311), (400), (422), (511), and (440), respectively, which were consistent with the reported data [34] (JCPDS No. 16-692). The XRD spectra of Fe_3_O_4_@MnPc-NDs were similar to that of Fe_3_O_4_ nanoparticles, indicating the functionalization of phthalocyanine did not affect the crystal structures of Fe_3_O_4_ nanoparticles. The functional groups on the surface were further characterized via infrared spectroscopy. As shown in Figure 4B, the characteristic peak at 579 cm^−1^ was ascribed to the Fe-O-Fe vibration in the Fe_3_O_4_ nanoparticles. The peaks at 3437, 1713, and 1613 cm^−1^ were assigned to the stretching vibrations of the O-H, C=O, and C=C, respectively. In addition, the peaks at 1393 and 1194 cm^−1^ were ascribed to the stretching vibrations of the C-N and C-O bonds, respectively. The magnetic properties of Fe_3_O_4_ nanoparticles and Fe_3_O_4_@MnPc-NDs were investigated using hysteresis loops. As indicated in Figure 4C, both Fe_3_O_4_ nanoparticles and Fe_3_O_4_@MnPc-NDs displayed obvious magnetic properties with saturation magnetization strengths of 49.46 and 36.37 emu/g, respectively. In addition, with the help of an external magnetic field, Fe_3_O_4_@MnPc-NDs dispersed in aqueous solution can be collected in a short time, indicating their excellent magnetism and ability for rapid magnetic separation.

### 3.2. Peroxidase-Mimicking Activity of Fe_3_O_4_@MnPc-NDs

**Figure 5:**
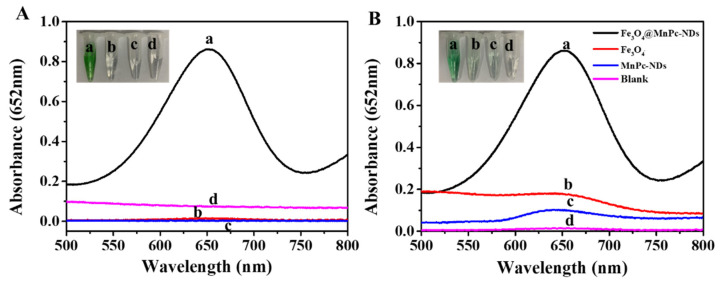
(**A**) UV–vis absorption spectra of (a) Fe_3_O_4_@MnPc-NDs + TMB + H_2_O_2_, (b) TMB + H_2_O_2_ (substrate control), (c) HAc-NaAc buffer (background control), (d) Fe_3_O_4_@MnPc-NDs (nanoparticle control); (**B**) Catalytic activity comparison of different materials in TMB + H_2_O_2_ system: (a) Fe_3_O_4_@MnPc-NDs, (b) Fe_3_O_4_ nanoparticles, (c) MnPc-NDs, (d) blank (no catalyst). Reaction conditions: [TMB] = 0.083 mM, [H_2_O_2_] = 1.67 mM.

The peroxidase-mimicking activity of Fe_3_O_4_@MnPc-NDs was evaluated with TMB and H_2_O_2_ as substrates. The absorption spectrum of the system was recorded through UV–vis absorption spectroscopy. As depicted in Figure 5A, only the “TMB + H_2_O_2_ + Fe_3_O_4_@MnPc-NDs” system exhibited an obvious absorption peak at 652 nm by comparing with the control groups, along with the appearance of a distinct blue color in solution. Moreover, the structural advantages of Fe_3_O_4_@MnPc-NDs were illustrated by comparing the catalytic activities of raw materials and composites. As shown in Figure 5B, the UV–visible spectrum of Fe_3_O_4_@MnPc-NDs exhibited a significant absorption peak at 652 nm. Moreover, the absorption intensity at 652 nm of Fe_3_O_4_@MnPc-NDs was higher than that of other control samples (Fe_3_O_4_ nanoparticles and MnPc-NDs), indicating the higher catalytic activity after functionalization.

### 3.3. Optimization of the Catalyzed Conditions

**Figure 6:**
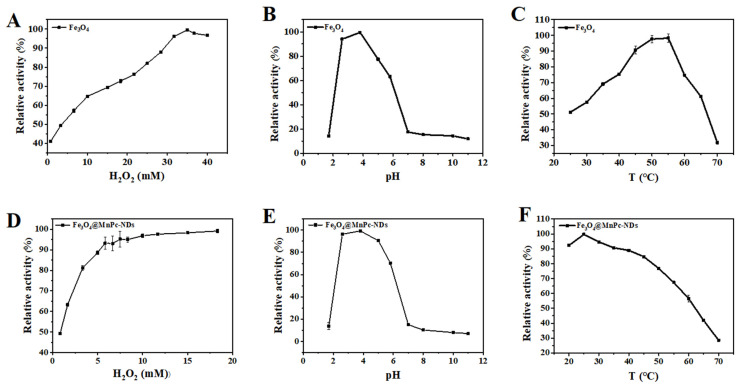
Peroxidase catalytic activity of Fe_3_O_4_-COOH nanoparticles under (**A**) varying hydrogen peroxide concentrations, (**B**) different pH values, and (**C**) varying temperatures. Peroxidase catalytic activity of Fe_3_O_4_@MnPc-NDs under (**D**) varying hydrogen peroxide concentrations, (**E**) different pH values, and (**F**) varying temperatures.

Similar to other peroxidase-like artificial enzymes, the catalytic activity of Fe_3_O_4_@MnPc-NDs might also depend on the H_2_O_2_ concentration, pH values, and temperature. Accordingly, the investigations were conducted to examine the catalytic activity of Fe_3_O_4_ nanoparticles and Fe_3_O_4_@MnPc-NDs under varying experimental conditions, specifically encompassing H_2_O_2_ concentrations ranging from 0.8 to 13 mM, pH values spanning from 1.7 to 11, and temperatures ranging from 20 to 70 °C. As depicted in Figure 6A,D, the catalytic activity of two materials started to increase and then leveled off with the increase in H_2_O_2_ concentration. The best catalytic activity was achieved at an H_2_O_2_ concentration of 10 mM, lower than that of bare Fe_3_O_4_ nanoparticles (255 mM). As shown in Figure 6E, Fe_3_O_4_@MnPc-NDs exhibited the highest activity at pH 3.8, which was similar to that of Fe_3_O_4_ nanoparticles (Figure 6B). Moreover, a temperature of 25 °C was found to be the most favorable for the catalytic performance of Fe_3_O_4_@MnPc-NDs (Figure 6F), while a higher temperature (55 °C) was required for Fe_3_O_4_ nanoparticles to realize the maximum catalytic activity (Figure 6C). Therefore, Fe_3_O_4_@MnPc-NDs exhibited the maximum catalytic activity under the milder conditions compared with that of bare Fe_3_O_4_ nanoparticles, and H_2_O_2_ concentration of 10 mM, pH 3.8, and temperature 25 °C were chosen as the most suitable conditions for studying the catalytic activity of Fe_3_O_4_@MnPc-NDs.

### 3.4. Steady-State Kinetic Determination of Fe_3_O_4_@MnPc-NDs

**Figure 7:**
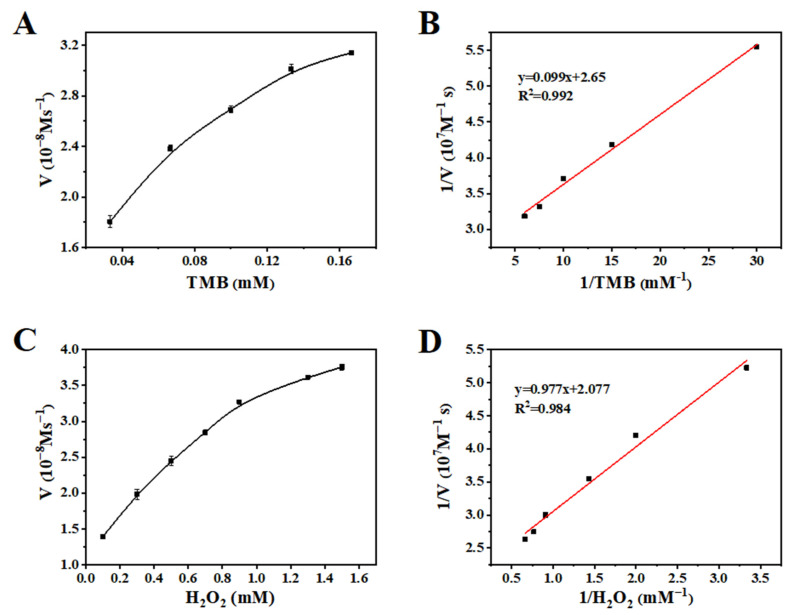
Steady-state kinetic assay of Fe_3_O_4_@MnPc-NDs: (**A**) 1.67 mM H_2_O_2_ with different TMB concentrations; and (**C**) 0.083 mM TMB with different H_2_O_2_ concentrations. Double-reciprocal plots are (**B**) and (**D**), corresponding to (**A**) and (**C**), respectively.

To further understand the catalytic mechanism of Fe_3_O_4_@MnPc-NDs, a series of kinetic assessments were performed by incrementally adjusting the concentration of one substrate while maintaining a constant level of the other substrate throughout the entire series of measurements. As displayed in Figure 7A,C, the typical Michaelis–Menten curves for TMB and H_2_O_2_ were measured within their appropriate concentration ranges. The corresponding relationships between the reciprocals of initial velocity and substrate concentration were obtained (Figure 7B,D). The oxidation reaction of Fe_3_O_4_@MnPc-NDs upon TMB and H_2_O_2_ conformed to the typical Michaelis–Mente equation. To assess the key kinetic attributes of the enzyme, including the Michaelis–Menten constant (K_m_) and the maximum initial reaction rate (V_max_), the Lineweaver–Burk plot was utilized (Table 1). K_m_ serves as an indicator of enzyme affinity for substrates. As the K_m_ value decreases, the affinity of the enzyme toward its substrate increases. Compared with HRP and Fe_3_O_4_ nanoparticles, Fe_3_O_4_@MnPc-NDs exhibited lower K_m_ values for H_2_O_2_, indicating that H_2_O_2_ can be detected at lower concentrations.

### 3.5. Detection of H_2_O_2_

**Figure 8:**
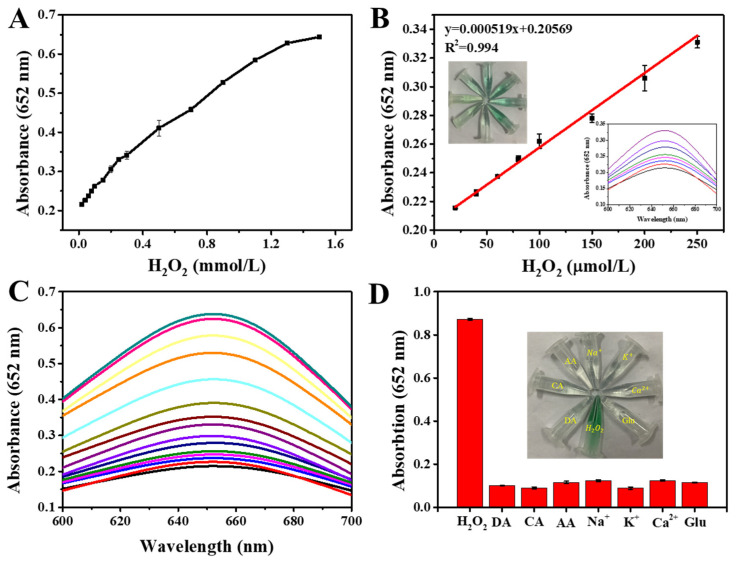
(**A**) UV–vis absorption spectra of ox-TMB at 652 nm varying with H_2_O_2_ concentrations; (**B**) the linear calibration curve of H_2_O_2_ (illustrated with photos of their UV–vis absorption spectra and corresponding color changes); (**C**) UV–vis absorption spectra of ox-TMB across varying H_2_O_2_ concentrations; (**D**) the UV–vis absorption spectra and corresponding color changes in the Fe_3_O_4_@MnPc-NDs sensing system ox-TMB in the presence of different substances including H_2_O_2_, dopamine (DA), citric acid (CA), ascorbic acid (AA), Na^+^, K^+^, Ca^2+^ and glucose.

Based on the peroxidase-mimicking properties of Fe_3_O_4_@MnPc-NDs, a simple colorimetric approach for the quantification of H_2_O_2_ was devised. As depicted in Figure 8A,C, a distinct enhancement in the absorbance of the mixture of Fe_3_O_4_@MnPc-NDs and TMB at 652 nm was observed, with the H_2_O_2_ concentration ranging from 0.2 to 15 mM. Based on the variation of absorbance and color change in solution, a linear calibration curve was obtained with the H_2_O_2_ concentration ranging from 20 to 250 μM (Figure 8B). According to the obtained linear equation, the detection limit (LOD) of H_2_O_2_ was calculated to be 4.7 μM, which was lower than the values previously reported in the literature (Table 2). In order to evaluate the influence of interfering substances, a range of potential interferences, including dopamine (DA), citric acid (CA), ascorbic acid (AA), glucose, as well as ions like Na^+^, K^+^, and Ca^2+^ were chosen. As depicted in Figure 8D, the solution color of Fe_3_O_4_@MnPc-NDs and TMB did not show any significant change after adding the interfering substances, even though their concentration was five times higher than that of H_2_O_2_, indicating the high selectivity and specificity for H_2_O_2_ detection.

### 3.6. Detection of AA and GSH

**Figure 9:**
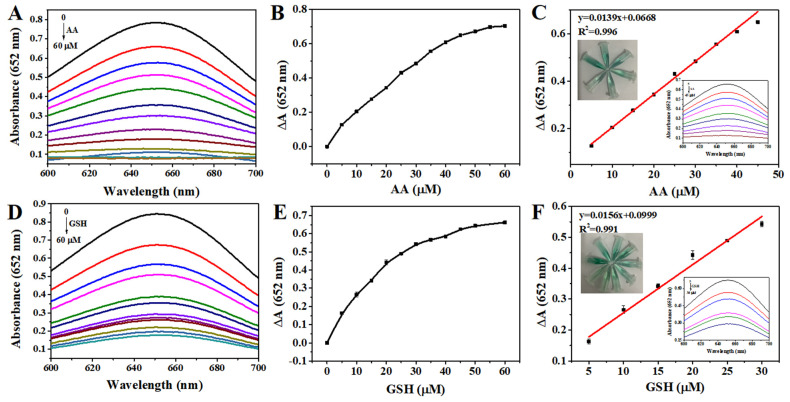
(**A**,**B**) UV–vis absorption spectra overlay of ox-TMB at different AA/GSH concentrations; (**C**,**D**) UV–vis absorption changes in ox-TMB at different AA/GSH concentrations at 652 nm; (**E**,**F**) linear calibration curve of AA/GSH (illustrated with photos of corresponding color changes and their UV–vis absorption spectra).

**Figure 10:**
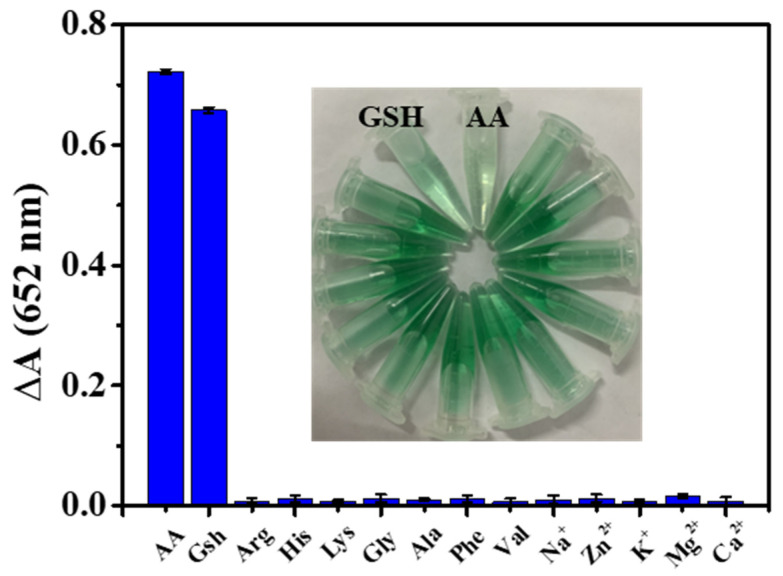
The UV–vis absorbance changes and corresponding color changes in the Fe_3_O_4_@MnPc-NDs sensing system ox-TMB in the presence of different substances including Arg (arginine), His (histidine), Lys (lysine), Gly (glycine), Ala (alanine), Phe (phenylalanine), Val (valine), Mg^2+^, Zn^2+^, Na^+^, K^+^ and Ca^2+^.

Based on the antioxidant effects of AA and GSH, a colorimetric approach for detection was established using the system of Fe_3_O_4_@MnPc-NDs and TMB. As shown in Figure 9A, the absorbance of the sensing platform at 652 nm exhibited a gradual decline upon the addition of ascorbic acid and GSH (Figure 9A,B), along with the fading of the blue solution. Based on the variation curve of absorbance at 652 nm upon the AA (Figure 9C) or GSH (Figure 9D) concentration, a linear equation was obtained for AA (ΔA = 0.0139 [AA] + 0.0668 (R^2^ = 0.996)) and GSH (ΔA = 0.0156 [GSH] + 0.0999 (R^2^ = 0.991)) (Figure 9E,F). According to these equations, the detection limit was calculated to be 0.161 μM for AA, and 0.188 μM for GSH, which were comparable to those reported by colorimetric methods (Table 3). Using Arg (arginine), Ala (alanine), His (histidine), Lys (lysine), Gly (glycine), Phe (phenylalanine), Val (valine), Mg^2+^, Zn^2+^, Na^+^, K^+^, and Ca^2+^ as interfering substances, the selectivity study for AA/GSH determination was conducted based on Fe_3_O_4_@MnPc-NDs. As shown in Figure 10, even though the concentrations of numerous interfering substances escalated to tenfold that of AA/GSH, the solution color of Fe_3_O_4_@MnPc-NDs/TMB/H_2_O_2_ system did not exhibit any significant change, along with negligible alterations in absorbance at 652 nm.

### 3.7. Detection of Ascorbic Acid and Glutathione in Actual Samples

To evaluate the practical application of this colorimetric method, the ascorbic acid content in vitamin C was tested. Specifically, vitamin C was diluted to a certain concentration and then added to the Fe_3_O_4_@MnPc-NDs/TMB/H_2_O_2_ system. To further corroborate the precision of this method, a known concentration of AA was introduced into the diluted sample of vitamin C tablets. As evident from Table S1, the recovered quantities of AA in these samples were extremely close to the expected values, with recovery rates higher than 98% and the relative standard deviation (RSD) less than 5%. The colorimetric method was also used to detect the content of glutathione in the purchased whitening serum. As shown in Table S2, the sample recoveries were higher than 99%, with an RSD of less than 5%, indicating the high accuracy of this method for the analysis of real samples.

### 3.8. Recyclability Testing

**Figure 11:**
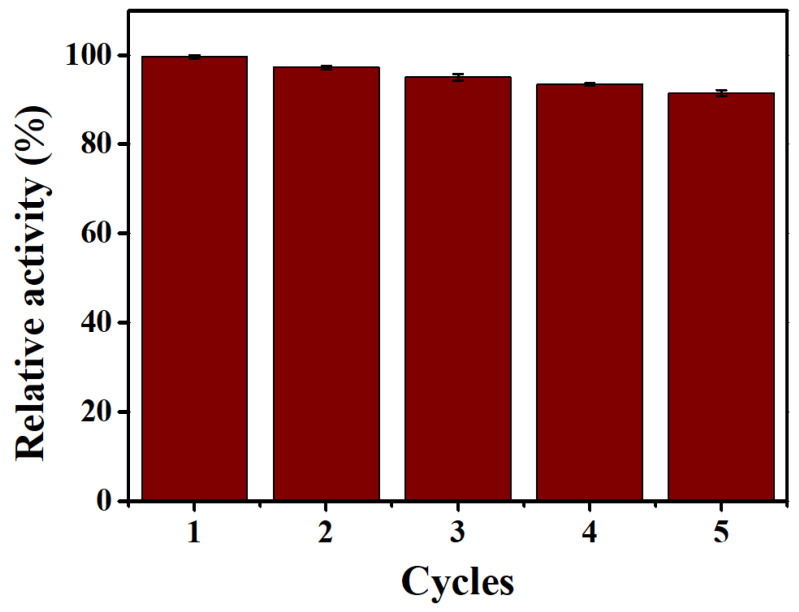
The selectivity analysis of AA detection with different interferents based on Co-POP + TMB system. The catalytic activity of the Fe_3_O_4_@MnPc-NDs in five successive recycling catalysis. (The conversion of the catalytic activity with the first run as 100%).

To evaluate the recyclability of Fe_3_O_4_@MnPc-NDs, the recovered Fe_3_O_4_@MnPc-NDs were added into the “TMB + H_2_O_2_” system, and their catalytic activity was tested. As shown in Figure 11, after five catalytic cycles, Fe_3_O_4_@MnPc-NDs still possessed high peroxidase activity with catalytic activity around 90%, indicating the good reusability of Fe_3_O_4_@MnPc-NDs.

## 4. Conclusions

In summary, the Fe_3_O_4_@MnPc-NDs composites were effectively synthesized via the hydrothermal method. Fe_3_O_4_@MnPc-NDs demonstrated enhanced peroxidase-like activity compared with the bare Fe_3_O_4_ nanoparticles, which could oxidize the colorless TMB to blue ox-TMB in the presence of H_2_O_2_. Based on the inhibitory effect of AA/GSH on this catalytic process, a highly sensitive and rapid colorimetric sensor was developed, capable of determining AA and GSH with detection limits of 0.161 and 0.188 μM, respectively. This developed sensor exhibited high selectivity and exceptional reproducibility, enabling its application in determining AA levels in vitamin C tablets and GSH concentrations in whitening serum, indicating the practical potential of Fe_3_O_4_@MnPc-NDs in colorimetric assays.

## Figures and Tables

**Table 1:** Comparison of apparent steady-state kinetic parameters between Fe_3_O_4_@MnPc-NDs and other artificial enzyme mimics.

Material	Catalytic Substrate	K_m_ (mM)	V_max_ (10^−8^ M S^−1^)	Reference
Fe_3_O_4_@MnPc-NDs	TMB	0.037	3.78	This work
	H_2_O_2_	0.47	4.82	
Fe_3_O_4_	TMB	0.098	3.44	[35]
	H_2_O_2_	154	9.78	
HRP	TMB	0.434	10	[35]
	H_2_O_2_	3.7	8.71	
Cu-Ag/rGO	TMB	8.61	7.02	[36]
	H_2_O_2_	0.634	4.26	

**Table 2:** Comparison of detection limit and linear range for detecting H_2_O_2_ using different peroxidase mimetic enzymes.

Material	Linear Range (μM)	Detection Limit (μM)	Reference
Fe_3_O_4_@MnPc-NDs	20–250	4.7	This work
Fe_3_O_4_	5–100	3	[37]
Copper	10–1000	10	[38]
Pt-MnO_2_/GOP	2–13,330	5	[39]
Au@Ag	10–10,000	6	[40]

**Table 3:** Comparison of linear range and detection limit for detecting AA and GSH using different peroxidase mimetics.

Catalyst	Detection	Linear Range (µM)	Detection Limit (μM)	Reference
N-CQDs	AA	5–40	1.77	[41]
N, S-CDs	AA	10–200	4.69	[42]
MIL-88	AA	2.57–10.10	1.03	[43]
Fe_3_O_4_@MnPc-NDs	AA	5–45	0.161	This work
QDs	GSH	5–250	0.6	[44]
Cd-Se/ZnS QDs	GSH	10–180	1.5	[45]
Co, N-HPC	GSH	0.05–30	0.036	[46]
Fe_3_O_4_@MnPc-NDs	GSH	5–30	0.188	This work

## Data Availability

The original contributions presented in this study are included in the article/Supplementary Materials. Further inquiries can be directed to the corresponding author.

## Supplementary Materials

The following supporting information can be downloaded at: https://www.mdpi.com/article/10.3390/molecules30071484/s1. Table S1 Recovery of AA from vitamin C samples by labeling. Table S2 Standard addition and recovery of GSH in whitening essence.
