# Supplementary material for: Manganese Phthalocyanine-Based Magnetic Core–Shell Composites with Peroxidase Mimetic Activity for Colorimetric Detection of Ascorbic Acid and Glutathione
Source: Molecules. 2025 Mar 27;30(7):1484. doi: 10.3390/molecules30071484 (PMC11990450; doi:10.3390/molecules30071484)
Supplement: Supplementary file 1 [file molecules-30-01484-s001.zip › molecules-3509241-supplementary.pdf]

Manganese Phthalocyanine-Based Magnetic Core–Shell Composites with Peroxidase Mimetic Activity for Colorimetric Detection of Ascorbic Acid and Glutathione

Junchao Qi, Long Tian, Yudong Pang and Fengshou Wu *

Hubei Key Laboratory of Novel Reactor and Green Chemical Technology, Key Laboratory of Novel Biomass-Based Environmental and Energy Materials in Petroleum and Chemical Industry, School of Chemical Engineering and Pharmacy, Wuhan Institute of Technology, Wuhan 430072, China

* Correspondence: wfs42@126.com

Materials and measurements

All reagents were purchased from commercial suppliers and used without further purification. All solvents were analytical pure unless otherwise noted. ferric chloride hexahydrate ($FeCl_3·6H_2O$),3,3′,5,5′-tetramethylbenzidine (TMB), ethylene glycol (EG), poly(4-styrenesulfonic acid-co- maleic acid, SS:MA=3:1) sodium salt (PSSMA 3:1, Mw 20000), acetic acid (HAc), sodium acetate (NaAc), 4-nitrophthalic anhydride, citric acid, manganese chloride, urea, ammonium molybdate tetrahydrate, nitrobenzene, sodium sulfide nonahydrate, N,N-Dimethylformamide (DMF), toluene, diethyl ether, sodium carbonate, tetramethylbenzidine, hydrogen peroxide, ascorbic acid, glutathione were obtained from Aladdin (Shanghai, China).

Scanning electron microscope (SEM) images were obtained using a JEOL630-F field-emission scanning electron microscopy. Transmission electron microscope (TEM) images were collected with a JEM-2100 TEM instrument. Powder X-ray diffraction (XRD) patterns were acquired at room temperature on a D8 Advance X-ray diffractometer (Bruker AXS Germany) with Cu Kr radiation, and the recording speed was 1° min^{-1}. X-ray photoelectron spectra (XPS) were performed on an ESCALAB multifunctional imaging electron spectrometer (Thermo). The Fourier transform infrared (FT-IR) spectra were measured at wavenumbers of 400-4000 cm-1 using a NICOLET6700 spectrometer (ABB Bomen Canada) with KBr pellets. Absorption spectra were obtained with a UV–vis spectrophotometer (Shimadzu).

Table S1 Recovery of AA from vitamin C samples by labeling.

AA Catalyst (μM)	AA additive quantity (μM)	observed value (μM)	Recovery rate (%, n=3)	Relative standard deviation (%, n=3)
18.93	5	23.68	98.96	2.93
	10	28.76	99.41	2.32
	15	33.44	98.56	1.59

Table S2 Standard addition and recovery of GSH in whitening essence.

GSH Catalyst	GSH additive	observed	Recovery	Relative

（μM）	quantity （μM）	value（μM）	rate（%, n=3）	standard deviation （%, n=3）
	3	19.49	99.01	0.98
16.67	6	22.53	99.38	0.71
	10	26.74	100.26	1.04
